# Incidence and impact of loss of pulsatility on outcomes after extracorporeal cardiopulmonary resuscitation – A retrospective cohort study

**DOI:** 10.1016/j.resplu.2026.101429

**Published:** 2026-07-24

**Authors:** Vinodh Bhagyalakshmi Nanjayya, Alistair Nichol, David M. Kaye, Lloyd Roberts, Kathleen Collins, Jayne Sheldrake, Alisa M. Higgins, Arne Diehl, Julia Coull, Aidan Burrell, Sacha Richardson, Judit Orosz, David Pilcher, Stephen Bernard, Paul Nixon, Vincent Pellegrino, D. James Cooper

**Affiliations:** a Department of Intensive Care and Hyperbaric Medicine, Alfred Hospital, Melbourne, VIC, Australia; b Australian and New Zealand Intensive Care Research Centre, Monash University, Melbourne, Australia; c School of Public Health and Preventive Medicine, Monash University, Melbourne, Australia; d Department of Cardiology, Alfred Hospital, Melbourne, VIC, Australia; e Australian and New Zealand Intensive Care Society Centre for Outcome and Resource Evaluation, Prahran, VIC, Australia

**Keywords:** Extracorporeal cardiopulmonary resuscitation, Refractory cardiac arrest, Out-of-hospital cardiac arrest, Loss of pulsatility, In-hospital cardiac arrest

## Abstract

**Aim:**

Loss of pulsatility, defined as zero pulse pressure, can occur following extracorporeal cardiopulmonary resuscitation (ECPR). The aim of this study was to determine the incidence of loss of pulsatility and assess its association with in-hospital mortality in ECPR patients.

**Methods and Settings:**

A retrospective cohort study of consecutive ECPR patients admitted between January 2011 and December 2024 to a high volume ECMO centre. Loss of pulsatility was defined as any documented pulse pressure (difference in systolic and diastolic arterial blood pressure) of 0 mmHg during the first 12 h following ECPR. Univariable and multivariable logistic regression models were used to identify association with in-hospital mortality.

**Results:**

During the study, 221 ECPR patients – mean age 50(±13.7) years, 165 (75%) males, met the inclusion criteria. At least one episode of loss of pulsatility occurred in 108 patients (49%). The loss of pulsatility group had a higher proportion of out-of-hospital cardiac arrest and longer arrest-to-ECMO time. In-hospital mortality was higher in the loss of pulsatility group (74% vs 48%; *p* < 0.01). After adjusting for location, first monitored rhythm, cause of cardiac arrest, and arrest-to-ECMO time, loss of pulsatility remained an independent predictor of mortality (OR 2.55 [95%CI 1.32–4.91]; *p* = 0.01). Each additional hour of loss of pulsatility was independently associated with an increased risk of mortality (OR 1.17 [95%CI 1.05–1.30]; *p* = 0.003).

**Conclusions:**

Loss of pulsatility is an important predictor of mortality after ECPR. Future studies should elucidate the underlying causes of loss of pulsatility and determine whether strategies to prevent or treat it improve patient outcomes.

## Highlights

• Loss of pulsatility occurs in nearly 50% of patients following ECPR.
• Loss of pulsatility is an independent predictor of death after ECPR.
• Longer loss of pulsatility incrementally increases the risk of mortality.
• Loss of pulsatility may assist in clinical risk-stratification.

## Graphical abstract

ECPR – Extracorporeal cardiopulmonary resuscitation, OHCA – Out-of-hospital cardiac arrest, IHCA – In-hospital cardiac arrest.

**Figure:**
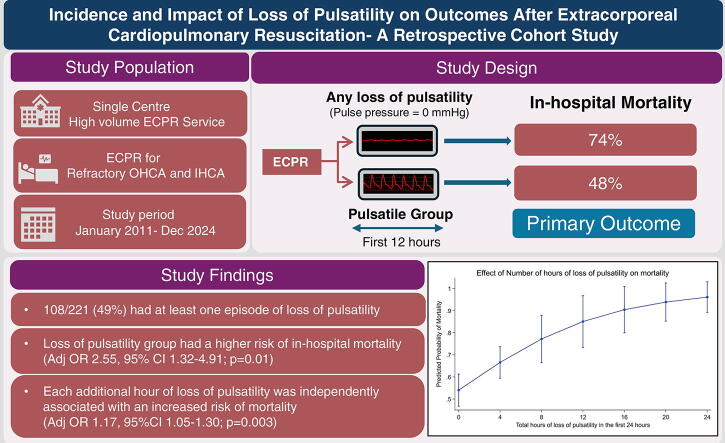

## Introduction

Extracorporeal cardiopulmonary resuscitation (ECPR) is a temporary cardiorespiratory support used in a selected group of patients with refractory out-of-hospital (OHCA) and in-hospital cardiac arrest (IHCA).^1^, ^2^, ^3^ It maintains oxygenation and perfusion while the patient recovers from the underlying cause of arrest and from systemic hypoxic-ischaemic injury. Overall ECPR survival in Australia is 27.5% for OHCA and 34.5% for IHCA.^4^, ^5^ Worldwide, ECPR survival ranges from 9% to 43% for OHCA and 20% to 34% for IHCA.^1^, ^2^

Following ECPR, some patients develop loss of pulsatility, a state of complete absence of pulsatility characterised by a flat arterial line trace on the monitor (arterial pulse pressure = 0 mmHg). This may persist despite measures to maintain contractility and optimise loading conditions. Prior research shows that low pulse pressure, defined as pulse pressure <15–20 mmHg before or after initiation of veno-arterial extracorporeal membrane oxygenation (VA ECMO) in cardiogenic shock, is associated with poor survival and increased risk of acute brain injury.^6^, ^7^ Similarly, ECPR studies show that patients with low pulse pressure fail to wean from VA ECMO and face a higher mortality risk.^8^, ^9^ However, the incidence of complete loss of pulsatility and its impact on outcomes after ECPR is currently unknown.

The aim of this study was to determine the incidence of loss of pulsatility and evaluate its impact on in-hospital outcomes following ECPR.

## Materials and methods

### Study design and setting

A retrospective, single-centre study was conducted at the Alfred Hospital, Melbourne, Australia, between January 2011 and December 2024. The Alfred Hospital is a high volume ECPR centre providing round-the-clock in-hospital ECPR for OHCA and IHCA, and a weekday prehospital ECPR service. Details of the ECPR program and management guidelines are provided in the Supplementary Appendix and Alfred ICU’s free online ECMO guidelines.^10^ It is also Victoria state’s referral centre for advanced mechanical cardiac support and heart and lung transplantation. Since 2019, the Alfred Hospital has been part of the Victorian ECMO Service (VECMOS), a state-wide network of hospitals and ambulance services providing ECMO.^11^ As part of VECMOS, ECPR patients were transferred from other centres for ongoing management, including ECMO weaning or consideration of ventricular assist device (VAD) insertion or heart transplantation.

All ECPR patients admitted to the Alfred Hospital between January 2011 and December 2024 were eligible for inclusion. The patients were identified from Alfred ICU’s local ECMO database.

### Data collection

Data on patient demographics, cardiac arrest characteristics, ECPR initiation, illness severity (APACHE-III-J score), coronary angiogram/intervention, organ supports, ICU and hospital lengths of stay, mortality, discharge destination, Left Ventricular Ejection Fraction (LVEF) at hospital discharge, and cerebral performance category (CPC) scores were extracted from the hospital medical records. Utstein definitions were used for collecting details of OHCA.^12^

All systolic and diastolic blood pressure measurements documented in the ICU monitoring chart during the first 24 h following ECPR initiation were reviewed, with hourly measurements extracted for the first 12 h and 4 h thereafter. Pulse pressure was calculated as the difference between arterial systolic and diastolic blood pressure. Patients with a pulse pressure of 0 mmHg at any hour in the first 12 h were classified as the loss of pulsatility group. Patients without any blood pressure measurements in the first 12 h were excluded. For patients with multiple ECPR episodes, only the first ECPR episode was included.

### Outcome measures

The primary outcome was in-hospital mortality. Secondary outcomes included ECMO duration, weaning from ECMO, ICU and hospital length of stay, VAD or heart transplantation, LVEF and CPC score at hospital discharge, and discharge destination.

### Statistical analysis

Continuous variables were summarised as mean (SD) or median (IQR) and compared using Student’s *t*-test or Wilcoxon rank-sum test; categorical variables as *n* (%) using Fisher’s exact test. A linear mixed-effects model with a random intercept per patient compared pulse pressure trajectories between groups. Multivariable logistic regression assessed the impact of loss of pulsatility on in-hospital mortality, with covariates selected based on clinical significance and published literature. Sensitivity analyses examined time-weighted mean pulse pressure and total hours of loss of pulsatility as alternative independent variables. To assess the impact of changes in clinical practice over time, patients were divided into three eras: prior to 2015, 2016–2019, and 2020–2024, with logistic regression and joint Wald tests used to assess the effect of era and its interaction with loss of pulsatility on mortality. A two-sided *p* ≤ 0.05 was considered statistically significant. Analyses were performed using StataSE 18 (StataCorp, College Station, TX, USA). Alfred Hospital’s HREC provided ethics approval with waiver of informed consent (Project number 334/19).

## Results

**Fig. 1:**
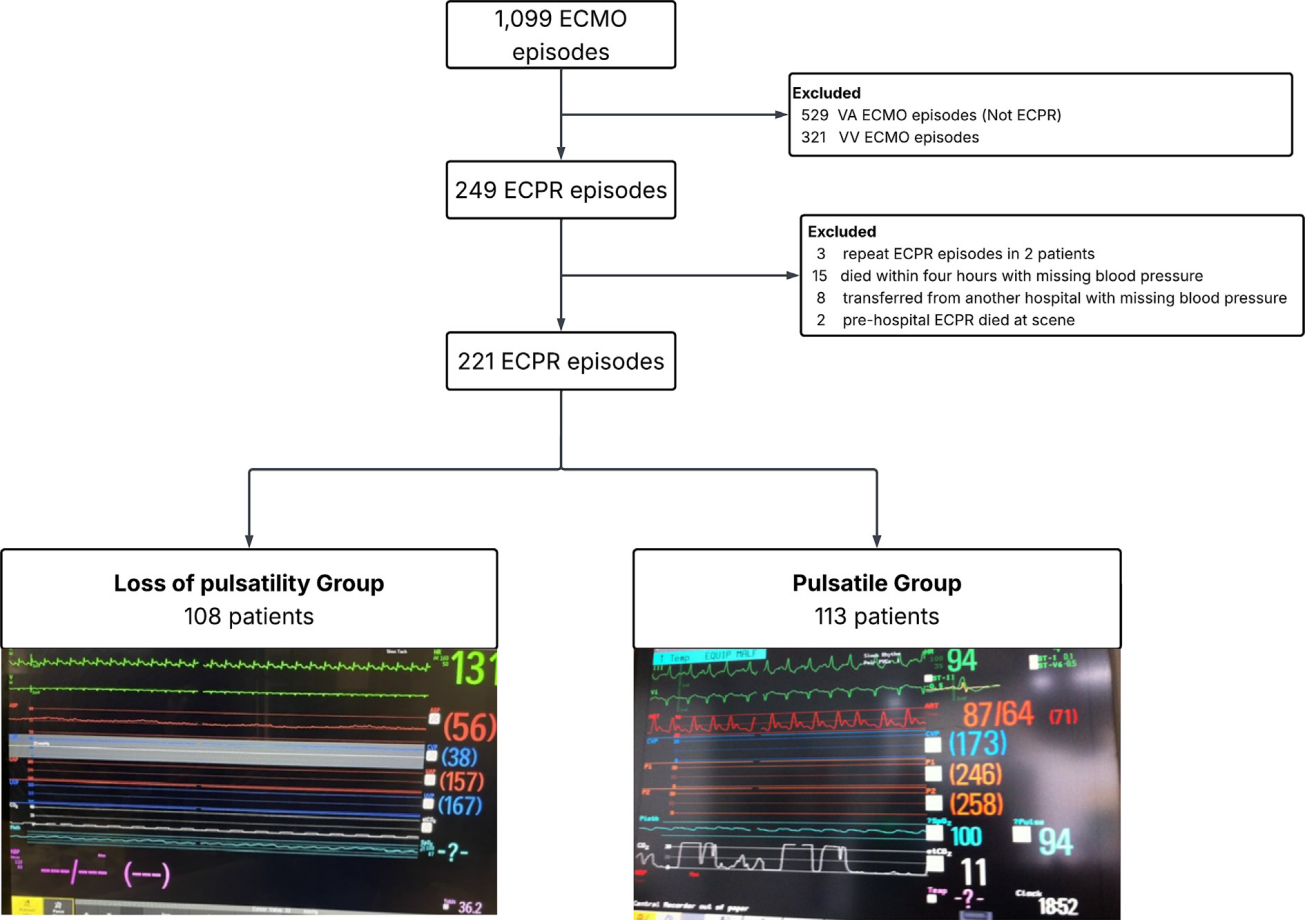
**ECMO – extracorporeal membrane oxygenation, VA – venoarterial, VV – venovenous, ECPR – extracorporeal cardiopulmonary resuscitation**.

During the study period, 246 patients underwent 249 ECPR episodes – one patient had three and another had two episodes. After excluding 25 patients – 17 who died within four hours and 8 transferred from other centres, both with no blood pressure documentation – 221 patients were included in the final analysis (Fig. 1).

The mean (SD) age was 50 (13.7) years, and 165 (75%) were male. Loss of pulsatility occurred in 108 (49%) patients, of whom 41 (38%) had loss of pulsatility at ECMO initiation. Median onset of loss of pulsatility was 2 (IQR 1–5) h and median duration 5 (IQR 2–9) h. Loss of pulsatility duration was 1 h in 29 (26.9%), 2–4 h in 24 (22.2%), 5–8 h in 28 (25.9%), and 9–12 h in 27 (25.0%) patients. Eight patients (7%) had persistent loss of pulsatility throughout the first 12 h; underlying causes included left main culprit lesion or dissection (*n* = 4), and refractory VF, severe myocarditis, end-stage dilated cardiomyopathy, and postoperative tamponade (one each).

**Fig. 2:**
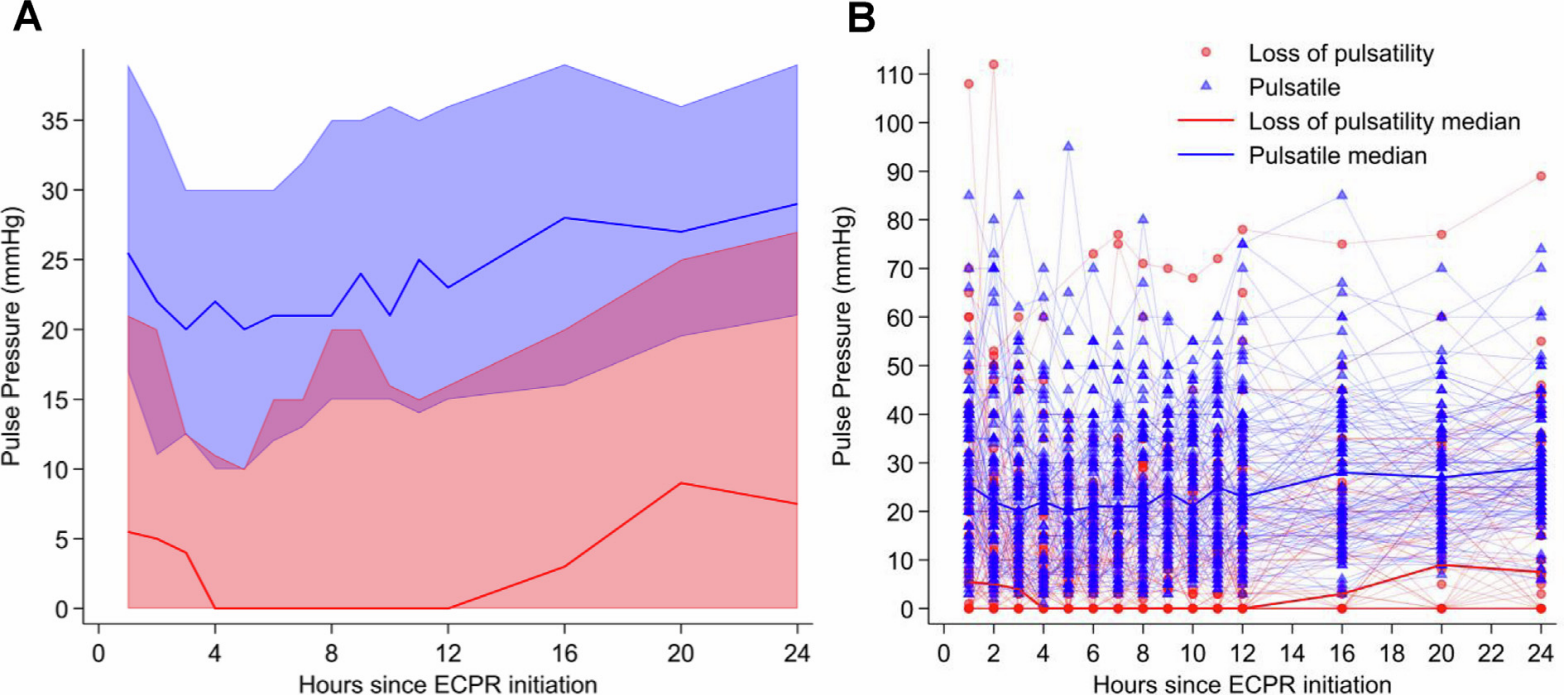
**Pulse pressure trajectories following ECPR initiation in the loss of pulsatility and pulsatile groups**. (A) The pulse pressure curves in patients in the loss of pulsatility and pulsatile groups. The pulse pressure curves (solid red and blue lines) show the hourly medians with the interquartile range represented by the shaded ribbon. The loss of pulsatility group (red) reached a nadir of 0 mmHg at 4 h following ECPR initiation, with gradual recovery after 12 h to reach its peak at 20–24 h. The pulsatile group (blue) maintained higher pulse pressure throughout the monitoring period. (B) The individual patient pulse pressure trajectories with group medians overlaid as thick lines. Of the 2436 total pulse pressure measurements possible during the study period, 2262 (93%) were available. ECPR – extracorporeal cardiopulmonary resuscitation. (For interpretation of the references to colour in this figure legend, the reader is referred to the web version of this article.)

Fig. 2 shows hourly pulse pressure distribution and individual trajectories between groups over the first 24 h. Median pulse pressure reached a nadir at 4 h after ECPR, recovering steadily from hour 12. Overall, the loss of pulsatility group had 14.2 mmHg lower pulse pressure than the pulsatile group across all repeated measurements (95% CI: 9.9–18.5 mmHg, *p* < 0.001) with considerable between-patient (SD 10.5 mmHg) and within-patient (SD 10.2 mmHg) variation. Time-weighted mean pulse pressure distribution is shown in Supplementary Appendix Fig. A1.

Table 1 summarises the baseline characteristics. Patients with loss of pulsatility were younger and more likely to have OHCA, with longer arrest-to-ECMO times, higher vasoactive inotropic scores (VIS), lower mean arterial pressure (MAP), and worse metabolic acidosis (mean [SD] base excess −21.6 [8.5] vs −16.9 [7.7], *p* = 0.003). Acute myocardial ischaemia was the predominant cause of cardiac arrest (51%), with the left anterior descending artery and its branches most implicated (48%). No significant differences were observed in witnessed arrest rates or initial rhythm among OHCA patients.

**Table 1:** Demographic and baseline characteristics of patients.

**Characteristic**	**All** **(*N* = 221)**	**Pulsatile group** **(*N* = 113)**	**Loss of pulsatility** **(*N* = 108)**	***p*-value**
Age (yrs), mean (SD)	49.8 (13.7)	52.8 (12.0)	46.7 (14.7)	<0.001
Males, no. (%)	165 (74.7)	85 (75.2)	80 (74.1)	0.88
BMI (kg/m^2^), mean (SD)	27.6 (5.9)	27.6 (5.9)	27.4 (5.9)	0.85
APACHE-III-J score, median (IQR)	117 (87–135)	113 (87–133)	119 (89–135.5)	0.62
**Co-morbidities, no. (%)**				
NYHA class IV	6 (2.7)	3 (2.7)	3 (2.8)	1.00
Diabetes	52 (23.5)	26 (23.0)	26 (24.1)	0.88
Chronic respiratory conditions	9 (4.1)	6 (5.3)	3 (2.8)	0.50
End stage renal failure	1 (0.5)	0 (0.0)	1 (0.9)	0.49
**Cardiac arrest details, no. (%)**				
OHCA	102 (46.2)	39 (34.5)	63 (58.3)	<0.001
Witnessed arrest*	83 (81.4)	34 (87.2)	49 (77.8)	0.30
Bystander CPR*	95 (93.1)	34 (87.2)	61 (96.8)	0.10
Shockable rhythm	115 (52.0)	54 (47.8)	61 (56.5)	0.23
Referred from other centres	51 (23.1)	26 (23.0)	25 (23.1)	1.00
Time from arrest to ECMO (min), median (IQR)	54 (36–84)	45 (30–64)	73 (41–99)	<0.001
**Cause of cardiac arrest, no. (%)**				
Acute myocadial ischaemia	113 (51.1)	55 (48.7)	58 (53.7)	0.38
Cardiomyopathy or myocarditis	15 (6.8)	8 (7.1)	7 (6.5)	
Primary arrhythmias	10 (4.5)	6 (5.3)	4 (3.7)	
PE/pulmonary hypertension	24 (10.9)	15 (13.3)	9 (8.3)	
Chronic rejection	3 (1.4)	3 (2.7)	0 (0)	
Peri-operative support	5 (2.3)	4 (3.5)	1 (0.9)	
Toxin related	6 (2.7)	2 (1.8)	4 (3.7)	
Miscellaneous**	45 (20.4)	20 (17.7)	25 (23.2)	
**Organ support details**				
ECMO blood flow at 4 h (L/min), mean (SD)	3.2 (0.8)	3.2 (0.7)	3.2 (0.8)	0.62
Mean arterial pressure† (mmHg), median (IQR)	67.9 (59.7–73.4)	70.7 (64.0–75.5)	65.7 (39.6–69.6)	<0.001
Vasoactive infusion‡, *n* (%)	212 (95.9)	107 (94.7)	105 (97.2)	0.5
Inotropic infusion‡, *n* (%)	192 (86.9)	93 (82.3)	99 (91.7)	0.05
Vasoactive inotropic score (VIS)‡, median (IQR)	37.5 (12.6–235.8)	27.5 (10.8–214.3)	41.5 (18.9–249.8)	0.06
Inotropic component of VIS, median (IQR)	6.2 (2.1–12.8)	3.2 (1.6–8.1)	9.8 (4.4–20.5)	<0.001
Mechanical ventilation, *n* (%)	221 (100%)	113 (100%)	108 (100%)	−
CRRT, *n* (%)	149 (67.4%)	70 (61.9%)	79 (73.1%)	0.09
**Arterial blood gas parameters during ECPR**				
pH, mean (SD)	6.95 (0.24)	7.01 (0.22)	6.88 (0.25)	<0.001
PaO_2_ (mmHg), median (IQR)	98 (58–338)	96 (57–172)	99 (59–476)	0.087
PaCO_2_ (mmHg), median (IQR)	50.5 (39–75)	51 (39–72)	50 (39–78)	0.71
Lactate (mmol/L), mean (SD)	10.3 (7.6–13.7)	9.6 (6.5–12.7)	11.1 (8.7–15.8)	<0.001
Base excess, mean (SD)	−19.2 (8.4)	−16.9 (7.7)	−21.6 (8.5)	<0.001
Bicarbonate (mmol/L), mean (SD)	12.8 (5.4)	14.0 (5.0)	11.6 (5.6)	0.002
**Coronary angiogram details**				
Coronary catheterisation, *n* (%)	131 (59.3)	63 (55.3)	68 (63.6)	0.22
Normal coronaries, *n* (%)	17 (13.1)	8 (12.7)	9 (13.4)	1.00
Coronary spasm, *n* (%)	9 (6.9)	4 (6.2)	5 (7.5)	1.00
Left main culprit, *n* (%)	20 (16.0)	6 (9.7)	14 (22.2)	0.086
LAD/branches culprit, *n* (%)	60 (48.0)	29 (46.8)	31 (49.2)	0.86
Left circumflex culprit, *n* (%)	23 (18.4)	11 (17.7)	12 (19.0)	1.00
Right coronary artery culprit, *n* (%)	25 (20.0)	14 (22.6)	11 (17.5)	0.51

SD – standard deviation, BMI – body mass index, IQR – interquartile range, APACHE – Acute Physiology And Chronic Health Evaluation, NYHA – New York Heart Association, OHCA – out-of-hospital cardiac arrest, CPR – cardiopulmonary resuscitation, PE – pulmonary embolism, ECMO – extracorporeal membrane oxygenation, IQR – interquartile range, VIS – vasoactive-inotropic score, CRRT – continuous renal replacement therapy, LAD – left anterior descending artery.

Data for BMI were missing for 10 (9%) in pulsatile group and 38 (35%) in loss of pulsatility group; on APACHE-III-J score for 2 (2%) and 4 (4%), respectively; on ECMO blood flow at 4 h 3 (3%) and 5 (5%), respectively; on arterial pH 10 (9%) and 10 (9%), respectively; on PaO_2_ 15 (13%) and 13 (12%), respectively; on PaCO_2_ 10 (9%) and 13 (12%), respectively; on arterial lactate 10 (9%) and 18 (17%), respectively; on base excess 12 (11%) and 13 (12%), respectively; on bicarbonate 12 (11%) and 13 (12%), respectively. All other variables were complete. Missing data were assumed to be missing at random and no imputation was performed.

* Calculated for patients with out-of-hospital cardiac arrest (*n* = 102).

** The miscellaneous group included 2 hypovolemia, 3 hypoxia, 1 anaphylaxis, 1 hypothermia, 3 coronary vasospasm, 1 sick sinus syndrome, 1 severe decompression sickness and 8 unknown causes in the pulsatile group. The loss of pulsatility group included 1 hypovolemia, 1 aortic aneurysm rupture, 2 Type A aortic dissections, 1 choriocarcinoma, 1 ruptured ectopic cancer, 1 catastrophic antiphospholipid antibody syndrome, 1 complete heart block, 2 drowning and 14 unknown causes of cardiac arrest.

† Time weighted mean in the first 24 h.

‡ Vasoactive infusions included both inotropes and vasopressors – vasopressors included noradrenaline and vasopressin and inotropes included adrenaline, milrinone and dobutamine. Vasoactive inotropic score was calculated using the following formula: *VIS = Dobutamine (µg/kg/min) × 1 + Adrenaline (µg/kg/min) × 100 + Noradrenaline(µg/kg/min) × 100 + Milrinone (µg/kg/min) × 10 + Vasopressin (µg/kg/min) × 10,000 + Levosimendan (µg/kg/min) × 50*. The maximum vasoactive inotropic score in the first 24 h is included in the analysis.

### Outcomes

Overall in-hospital mortality was 61% (72% OHCA, 51% IHCA) and was significantly higher in the loss of pulsatility group (80 [74.1%] loss of pulsatility vs 57 [47.8%] pulsatile group; *p* < 0.001) (Table 2). No significant difference in the causes of death was noted between the groups with neurological causes accounting for the majority of deaths (51.2% loss of pulsatility group vs 66.7% pulsatile group). The loss of pulsatility group had shorter ECMO duration and ICU and hospital lengths of stay, though durations were similar amongst survivors. They were less likely to wean from VA ECMO (74 [68.5%] loss of pulsatility vs 41 [36.3%] pulsatile group; *p* < 0.01). The LV decompression and VAD insertion rates were similar between groups. Two patients in the loss of pulsatility group needed heart transplantation versus none in the pulsatile group. Only 1 of the 8 patients with persistent loss of pulsatility survived. Discharge CPC, LVEF and discharge destination did not differ significantly amongst survivors.

**Table 2:** Primary and secondary outcomes.

**Outcome**	**Total** **(*N* = 221)**	**Pulsatile group** **(*N* = 113)**	**Loss of pulsatility** **(*N* = 108)**	***p*-value**
**Primary outcome**				
In-hospital mortality, no. (%)	134 (60.6)	54 (47.8)	80 (74.1)	<0.001
**Secondary outcomes**				
ECMO support days, median (IQR)	4.0 (1.2–6.9)	4.5 (2.4–8.1)	2.8 (0.9–6.0)	<0.001
ECMO support days in survivors, median (IQR)*	5.5 (4.0–9.0)	5.2 (4.0–9.0)	5.8 (4.1–8.3)	0.49
Successful weaning from ECMO, no. (%)	106 (48.0)	72 (63.7)	34 (31.5)	<0.001
ICU length of stay (days), median (IQR)	7.1 (1.5–16.1)	10.0 (3.9–18.7)	3.0 (0.6–11.2)	<0.001
ICU length of stay (days) in survivors, median (IQR)*	15.0 (9.6–26.1)	13.6 (9.9–29.7)	15.6 (9.3–21.6)	0.99
Hospital length of stay (days), median (IQR)	11.1 (2.2–29.7)	17.1 (7.0–32.7)	4.2 (1.0–21.9)	<0.001
Hospital length of stay (days) in survivors, median (IQR)*	30.8 (19.3–45.4)	31.6 (19.3–45.9)	30.8(20.3–45.3)	0.99
LV vent insertion, no. (%)	14 (6.3)	5 (4.4)	9 (8.3)	0.28
**Cause of death**				
Brain death	23 (17.2%)	11 (20.4%)	12 (15.0%)	0.32
HIE or ICH	54 (40.3%)	25 (46.3%)	29 (36.2%)	
Refractory cardiogenic shock	38 (28.4)	11(20.4)	27 (33.8)	
Other causes	19 (14.2%)	7 (13.0%)	12 (15.0%)	
Bridged to VAD, no. (%)	10 (4.5)	4 (3.5)	6 (5.6)	0.53
Survivors bridged to heart transplantation, no. (%)*	2 (2.3)	0 (0.0)	2 (7.1)	0.10
Survival with VAD/heart transplantation, no. (%)*	10 (11.5)	4 (6.8)	6 (21.4)	0.07
LVEF(%) at the time of discharge, median (IQR)*	55 (45–60)	55 (45–62)	55 (40–60)	0.40
CPC of survivors, no. (%)*				
1	72 (82.8)	49 (83.1)	23 (82.1)	0.35
2	11 (12.6)	7 (11.9)	4 (14.3)	
3	3 (3.5)	3 (5.1)	0 (0)	
4	1 (1.2)	0 (0)	1 (3.6)	
Discharge destination, no. (%)*				
Home	49 (56.3)	33 (55.9)	16 (57.1)	0.36
Rehabilitation	19 (21.8)	11 (18.6)	8 (28.6)	
Other hospital	17 (19.5)	14 (23.7)	3 (10.7)	
Neuro-rehabilitation	2 (2.3)	1 (1.7)	1 (3.6)	

ECMO – extracorporeal membrane oxygenation, IQR-interquartile range, ICU – intensive care unit, LV – left ventricle, HIE – hypoxic-ischaemic encephalopathy, ICH – intracerebral haemorrhage, VAD – ventricular assist device, LVEF – left ventricular ejection fraction, CPC – cerebral performance category score.

Data on LVEF at the time of discharge were missing for 17 (29%) survivors in the pulsatile group and for 5 (18%) in the loss of pulsatility group.

* Calculated for 59 survivors in the pulsatile group and 28 survivors in the loss of pulsatility group.

On multivariable logistic regression (Table 3), loss of pulsatility was an independent predictor of mortality after adjusting for age, sex, arrest location, initial rhythm, cardiac arrest cause, and arrest-to-ECMO time (Adjusted OR [95% CI]: 2.55 [1.32–4.91]; *p* = 0.005).

**Table 3:** Factors associated with in-hospital mortality.

**Characteristic**	**Univariable analysis**		**Multivariable analysis**	
	**OR (95% CI)**	***p*-value**	**Adj OR (95%CI)**	***p*-value**
**Loss of pulsatility**				
No	Ref		Ref	
Yes	3.12 (1.77–5.50)	<0.001	2.55 (1.32–4.91)	0.005
Age (yrs)	0.99 (0.97–1.01)	0.50	1.00 (0.98–1.03)	0.92
**Sex**				
Female	Ref		Ref	
Male	1.00 (0.54–1.85)	0.99	0.76 (0.35–1.64)	0.48
APACHE-III-J	1.00(0.996–1.01)	0.44	–	
**Location of cardiac arrest**				
OHCA	Ref		Ref	
IHCA	0.42 (0.24–0.73)	0.002	0.69 (0.32–1.48)	0.34
**First monitored rhythm**				
PEA/Asystole	Ref		Ref	
VT/VF	0.81 (0.47–1.40)	0.33	0.36 (0.17–0.76)	0.007
**Diagnostic group**				
Non-AMI	Ref		Ref	
AMI	1.77 (1.03–3.05)	0.04	2.24 (1.07–4.66)	0.03
Arrest to ECMO time (min)	1.02 (1.01–1.03)	<0.001	1.01 (1.00–1.02)	0.04
VIS*	1.005 (1.002–1.007)	<0.001	1.00 (1.00–1.01)	<0.001
pH†	0.06 (0.02–0.22)	<0.001	–	
PaO_2_ (mmHg)	1.00(1.00–1.002)	0.36	–	
PaCO_2_ (mmHg)	1.02 (1.01–1.03)	0.004	–	
Lactate (mmol/L)†	1.17 (1.09–1.25)	<0.001	–	
Base excess†	0.94 (0.90–0.97)	0.001	–	
**LV Vent**				
No	Ref			
Yes	0.86 (0.29–2.56)	0.78	–	
**VAD or heart transplant**				
No	Ref			
Yes	0.06 (0.01–0.46)	0.007	–	
**Continuous renal replacement therapy**				
No	Ref			
Yes	1.62 (0.91–2.87)	0.098	–	

OR – odds ratio, CI – confidence interval, APACHE – Acute Physiology and Chronic Health Evaluation, OHCA – out of hospital cardiac arrest, IHCA – in-hospital cardiac arrest, PEA – pulseless electrical activity, VT/VF – ventricular tachycardia/ventricular fibrillation, AMI – acute myocardial infarction, ECMO – extracorporeal membrane oxygenation, VIS – vasoactive inotropic support, LV – left ventricle, VAD – ventricular assist device.

The multivariable logistic regression showed adequate discrimination and calibration (area under receiver operating characteristic curve = 0.79, Hosmer-Lemeshow Goodness of Fit *χ*^2^ (8 df) = 5.77, *p* = 0.67).

* Maximum VIS in the first 24 h is included in the model.

† The blood gas parameters were not included in the final model due to significant correlation with the arrest-to-ECMO-time.

### Sensitivity analysis

**Fig. 3:**
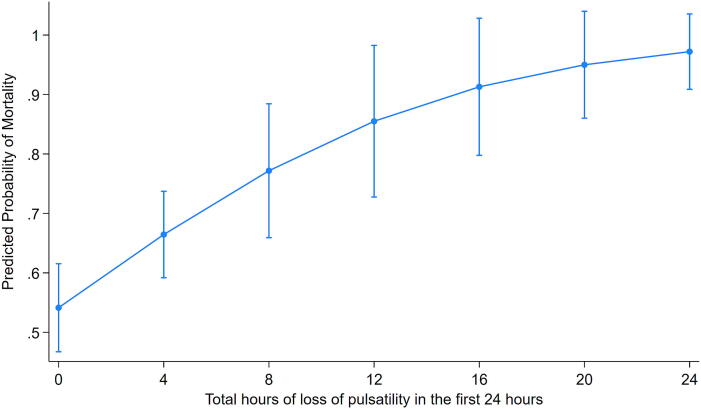
**The covariate-adjusted predicted probability of mortality across the duration of loss of pulsatility derived from the multivariable model shown in**Supplementary Appendix Table A2**. Error bars indicate 95% confidence interval**.

Each additional hour of loss of pulsatility was associated with a 17% (95% CI: 5–30%; *p* = 0.004) increase in the odds of in-hospital mortality (Supplementary Table A1, Fig. 3). A duration of loss of pulsatility exceeding 1.5 h best discriminated mortality from survival (Youden’s *J* = 0.305, sensitivity 0.48, specificity 0.83, AUC 0.65), indicating fair discriminative performance.

Each mmHg increase in time-weighted mean pulse pressure was associated with a 5% (95% CI: 2–7%) reduction in the odds of in-hospital mortality (Supplementary Appendix Table A2 and Fig. A2). Pulsatile patients were subdivided into normal pulsatility (time-weighted pulse pressure >20 mmHg, *n* = 75) and low pulsatility (≤20 mmHg, *n* = 38) subgroups. Mortality increased stepwise across the three groups: 38.7%, 65.8%, and 74.1% for normal pulsatility, low pulsatility, and loss of pulsatility, respectively (Fisher’s exact *p* < 0.001), with each stepwise decrease in pulsatility associated with a 2.12-fold increase in the odds of death (95% CI: 1.54–2.92, *p* < 0.001), further supporting a dose–response relationship between pulsatility reduction and mortality.

The proportion of loss of pulsatility patients declined markedly across the three eras-prior to 2015 (42/56,75%) 2016–2019 (40/70,57%), and 2020–2024(26/95, 27%) – while the mortality differences between the two groups remained consistent, with no significant era effect (Wald test, 2 df, *p* = 0.31) or group and time interaction (Wald test, 2 df, *p* = 0.18), suggesting that loss of pulsatility remains a consistent predictor of mortality regardless of era.

## Discussion

In this study, loss of pulsatility occurred in approximately 50% of ECPR patients, with 7% experiencing persistent loss of pulsatility throughout the first 12 h. Loss of pulsatility was an independent predictor of in-hospital mortality, with shorter VA ECMO duration, and lower weaning rates compared to the pulsatile group, and a dose–response relationship between increasing duration of loss of pulsatility and mortality risk.

In VA ECMO, pulsatility arises from the complex interplay between ventriculo-arterial and ECMO-arterial coupling.^13^ As a surrogate marker of cardiac output, it reflects both LV contractility and loading conditions.^14^ Post-arrest LV dysfunction arises from the precipitating aetiology (e.g., myocardial infarction) and post-resuscitation myocardial dysfunction.^15^, ^16^, ^17^ Prolonged CPR may further impair LV filling through CPR-related mechanical complications – including haemorrhagic shock, tamponade, and tension pneumothorax – as well as global ischaemia–reperfusion injury.^17^, ^18^, ^19^

VA ECMO flow further alters loading conditions. It reduces LV preload through diversion of venous return from the native heart.^20^ In addition, afterload may be elevated by vasoactive agents and the ECMO-native circulation interaction, though the extent to which VA ECMO itself contributes to afterload elevation remains inconsistent across studies.^13^, ^15^, ^20^, ^21^, ^22^ LV distension syndrome represents another recognised mechanism of reduced pulsatility, to which ECPR patients are particularly susceptible.^23^, ^24^ While LV venting rate of 6.3% in our cohort was comparable to other studies and did not differ significantly between groups, venting rate alone may not fully capture the burden of LV distension syndrome.^24^, ^25^ Nonetheless, the loss of pulsatility group had a longer arrest-to-ECMO duration and more profound acidosis which likely predisposed them to develop loss of pulsatility, and may itself account for the excess mortality observed in this group.

The excess mortality observed in the loss of pulsatility group may therefore reflect loss of pulsatility as a marker of whole-body hypoxic-ischaemic injury or myocardial injury severity rather than a direct cause of death. These patients had longer arrest-to-ECMO times, greater metabolic acidosis, higher lactate levels, and higher vasoactive medication requirements, indicating more severe myocardial dysfunction due to global hypoperfusion prior to ECMO initiation. The duration of loss of pulsatility may similarly reflect hypoxic-ischaemic burden, with longer duration representing greater cumulative injury-consistent with the observed dose–response relationship between duration and mortality. The non-significant increase in CRRT use further supports a higher hypoxic-ischaemic burden, though insufficient power may have precluded detection of a significant difference in this secondary outcome. Whether loss of pulsatility independently mediates mortality through other mechanisms warrants prospective investigation with mechanistic endpoints.

Approximately 70% of patients with loss of pulsatility failed to wean from VA ECMO, and 21% of survivors required bridging to VAD or heart transplantation – more than three times the rate observed in the pulsatile group – a clinically meaningful difference despite not reaching statistical significance. Some ECPR patients sustain significant myocardial injury or refractory VT necessitating bridging to VAD or heart transplantation. Loss of pulsatility may serve as an early predictor to guide timely consideration of these advanced therapies. Centres without VAD or heart transplant programmes may consider early referral of patients with loss of pulsatility to specialist centres with access to durable mechanical cardiac support.

Low pulsatility is an established marker of poor outcomes in VA ECMO patients.^6^, ^7^, ^8^ The SAVE score identified pre-ECMO pulse pressure below 20 mmHg as an independent mortality risk factor.^6^ However, that model did not assess the impact of loss of pulsatility after VA ECMO initiation. An agreed approach to capturing and reporting pulse pressure and loss of pulsatility following VA ECMO initiation could improve risk stratification and inform future prediction models.

Shou et al. looked at the impact of early low pulse pressure (<20 mmHg) within the first 12 h of VA ECMO initiation.^7^ They found that patients with low pulse pressure were at significantly higher risks of ischemic stroke, hypoxic-ischemic brain injury, seizures, intracranial haemorrhage, cerebral oedema and brain death. Approximately 19% of their cohort comprised ECPR patients. In contrast, our study did not find any significant difference in rates of brain death, hypoxic-ischaemic injury or intracranial haemorrhage between the pulsatile and loss of pulsatility groups.

Lee et al. demonstrated that low pulse pressure (<15 mmHg) for more than 6 h within the first 24 h after ECPR was significantly associated with failure to wean from VA ECMO, with all 16 patients who failed to wean subsequently dying.^8^ In contrast, our study specifically examined complete loss of pulsatility documented at any point during the first 12 h, finding significant association with both failure to wean and mortality. A further ECPR study found that the lower pulse pressure correlated with higher mortality, shorter ECMO duration, and reduced ICU and hospital length of stay, with mean pulse pressure within the first 24 h emerging as an independent predictor of survival.^9^ Our findings are consistent with this, finding a significant association between time-weighted mean pulse pressure and mortality.

Loss of pulsatility has been variably defined across studies, with thresholds ranging from pulse pressure below 15–20 mmHg. Our definition-complete equalisation of systolic and diastolic pressures-represents true loss of pulsatility, which may reflect an extreme degree of LV systolic impairment unable to generate any cardiac output, and may serve as an easily identifiable marker of myocardial injury severity following ECPR at the bedside. Future studies may consider incorporating absolute loss of pulsatility as a key prognostic variable after ECPR.

### Strengths and limitations

All ECMO data were prospectively collected in the local ECMO database, minimising missing and recording bias. Closely monitored blood pressure measurements enabled precise temporal analysis. The sample size allowed adjustment for multiple covariates in the multivariable analyses. The objective definition of loss of pulsatility and outcome measures minimised misclassification risk. Multiple analytical methods yielded consistent results, underscoring the robustness of our findings, and temporal analysis accounted for practice change over the 14-year study period.

The single-centre, retrospective study design limits external validity and introduces potential selection bias. Blood pressure measurements were collected from ICU monitoring chart documentation, and transient episodes of loss of pulsatility may have been missed if not recorded at the time of occurrence, potentially underestimating its prevalence and impact on mortality. Prospective studies utilising continuously collected electronic data may allow more complete capture of loss of pulsatility. Residual confounding due to temporal changes in clinical practice and variability in the management of loss of pulsatility and LV decompression cannot be fully excluded. The retrospective design also precluded precise determination of the underlying cause of loss of pulsatility in individual patients, and absence of contemporaneous echocardiography further limited characterisation of cardiac contractility during these events; prospective studies incorporating systematic echocardiographic assessment may provide mechanistic insights. Finally, missing data were assumed to be missing at random and no imputation was performed.

## Conclusion

Loss of pulsatility in the first 12 h after ECPR is an independent predictor of mortality and the mortality risk increases with the duration of loss of pulsatility. Future studies should delineate the mechanisms and risk factors contributing to loss of pulsatility and determine whether strategies to prevent or reverse it improve patient outcomes.

## CRediT authorship contribution statement

**Vinodh Bhagyalakshmi Nanjayya:** Conceptualization, Data curation, Formal analysis, Investigation, Methodology, Project administration, Software, Validation, Writing – original draft, Writing – review & editing. **Alistair Nichol:** Conceptualization, Methodology, Resources, Supervision, Validation, Visualization, Writing – original draft, Writing – review & editing. **David M. Kaye:** Conceptualization, Methodology, Resources, Supervision, Validation, Visualization, Writing – original draft, Writing – review & editing. **Lloyd Roberts:** Investigation, Resources, Writing – review & editing. **Kathleen Collins:** Data curation, Resources, Writing – review & editing. **Jayne Sheldrake:** Data curation, Resources, Writing – review & editing. **Alisa M. Higgins:** Conceptualization, Methodology, Resources, Supervision, Visualization, Writing – original draft, Writing – review & editing. **Arne Diehl:** Data curation, Investigation, Resources, Writing – review & editing. **Julia Coull:** Investigation, Resources, Writing – review & editing. **Aidan Burrell:** Investigation, Resources, Writing – review & editing. **Sacha Richardson:** Investigation, Resources, Writing – review & editing. **Judit Orosz:** Investigation, Resources, Writing – review & editing. **David Pilcher:** Investigation, Resources, Writing – review & editing. **Stephen Bernard:** Investigation, Resources, Writing – review & editing. **Paul Nixon:** Investigation, Resources, Writing – review & editing. **Vincent Pellegrino:** Data curation, Investigation, Resources, Writing – review & editing. **D. James Cooper:** Conceptualization, Investigation, Methodology, Resources, Supervision, Validation, Visualization, Writing – original draft, Writing – review & editing.

## Funding

Not applicable. The study did not receive any funding.

## Data availability statement

The de-identified participant data that support the findings of this study may be made available from the corresponding author on reasonable request and subject to institutional approvals.

## Declaration of competing interest

VBN is supported by the Commonwealth through an Australian Government Research Training Program Scholarship [https://doi.org/10.82133/C42F-K220]. AMH is supported by an NHMRC Investigator Grant (GNT 2008447). AB is supported by MRFF investigator (201110) and Heart Foundation grants (105213). DJC is supported by an NHMRC Investigator Grant (GNT 2016324).

All the other authors have no conflict of interests to declare.

## Appendix A Supplementary material

Supplementary Data 1 Supplementary Appendix: Additional details on the Alfred ICU’s ECPR management guidelines and tables and figures from the secondary analysis.
