# Supplementary material for: Incidence and impact of loss of pulsatility on outcomes after extracorporeal cardiopulmonary resuscitation – A retrospective cohort study
Source: Resusc Plus. 2026 Jul 24;31:101429. doi: 10.1016/j.resplu.2026.101429 (PMC13471356; doi:10.1016/j.resplu.2026.101429)
Supplement: Supplementary file 1 [file mmc1.docx]

# Supplementary Appendix

**Incidence and Impact of Loss of Pulsatility on Outcomes After Extracorporeal Cardiopulmonary Resuscitation- A Retrospective Cohort Study**

Vinodh Bhagyalakshmi Nanjayya FCICM, DDU, Mstr Biostat^1,2,3^, Alistair Nichol FCICM, PhD^1,2,3^, David M Kaye FRACP, PhD^4^, Lloyd Roberts FRACP, FANZCA, FCICM^1,3^, Kathleen Collins BN, MPH^1^, Jayne Sheldrake^1^, Alisa M Higgins MPH, PhD^2,3^ , Arne Diehl FACEM, FCICM^1,3^, Julia Coull FCICM^1,3^, Aidan Burrell FCICM^1,2,3^, Sacha Richardson FCICM^1,3^, Judit Orosz FCICM, DDU, MPH^1,3^, David Pilcher FCICM^1,2,3,5^, Stephen Bernard FACEM, FCICM, MD^1,3^, Paul Nixon FCICM, FRACP^1,3^, Vincent Pellegrino FCICM^1,2,3^, D James Cooper BMBS, MD, FCICM^1,2,3^

Affiliation:

1. Department of Intensive Care and Hyperbaric Medicine, Alfred Hospital, Melbourne, VIC, Australia
2. Australian and New Zealand Intensive Care Research Centre, Monash University, Melbourne, Australia
3. School of Public Health and Preventive Medicine, Monash University, Melbourne, Australia
4. Department of Cardiology, Alfred Hospital, Melbourne, VIC, Australia
5. Australian and New Zealand Intensive Care Society Centre for Outcome and Resource Evaluation, Prahran, VIC, Australia

Corresponding author’s contact:

Vinodh B Nanjayya

Department of Intensive Care and Hyperbaric Medicine,

541 St Kilda Road, Melbourne, VIC 3004

E-mail: v.nanjayya@alfred.org.au

**Brief Description of the Alfred Hospital Extracorporeal cardiopulmonary resuscitation (ECPR) program (Source:** [Introduction – Alfred ECMO Guideline](https://ecmo.icu/ecpr-introduction/?parent=menuautoanchor-27&def=true)**)**

The Alfred Hospital ECPR program started in 2011 as part of a pilot research study. The service provides round-the-clock ECPR for both in-hospital and out-of-hospital refractory cardiac arrests. Pre-hospital ECPR provision was added in 2020 as a weekday 9AM-5 PM service.

The current eligibility criteria are as follows:

**General Inclusion Criteria***

•	Combined age and arrest-to-ED arrival time (min) below 100 (ambulance call time to time of decision in min) **, alternatively arrest-to-ED arrival time only <60min

•	Witnessed cardiac arrest

•	Shockable initial rhythm

•	Bystander CPR within 5 min

•	No known end-stage disease

* Does not apply to accidental hypothermia

** Return of spontaneous circulation (ROSC) <20 min is considered continuous cardiac arrest; ROSC >20 min is regarded as separate arrests; the longer cardiac arrest time is taken

Factors that may also form part of the decision-making at the bedside and favour inclusion include signs of life such as movements, breathing effort, gasping, and any ROSC period during the arrest (only ROSC for 20min or greater would be considered separate arrest episodes)

Factors at the bedside that are usually considered as exclusion are age >65 years, end-tidal CO_2_ less than 10 mmHg, femoral cannulation impossible (e.g. iliaco-femoral occlusion / occluded IVC filter / severe peripheral vascular disease), known more than mild aortic regurgitation, presence of pericardial effusion or tamponade with suspected aortic dissection.

*Special circumstances*

Include arrests in the coronary catheterisation laboratory, accidental hypothermia and selected toxicology cases.

Please refer to the online guidelines for more information on the definitions and special circumstances.

Trained ICU consultants initiate VA ECMO using ultrasound-guided percutaneous ECMO cannulation techniques. Trained registered nurses provide 1:1 nurse-to-patient bedside care with support from extracorporeal life support (ECLS) nurses. In addition to the twice daily ICU consultant rounds, a senior ICU ECMO specialist performs an ECMO-specific daily ward round. Cardiohelp (Getinge, Rastatt, Germany), Rotaflow II (Getinge, Gothenberge, Sweden) and Rotaflow (Maquet, Rastatt, Germany) pumps were used to manage patients. Patients who could not be weaned from VA ECMO, if eligible for durable LVAD support, receive HeartMate 3^TM^ LVAD (Abbott, Pleasanton, CA) as a bridge to heart transplantation (in Australia, durable LVAD implantation was only compensable for use as a bridge to heart transplantation). If clinically indicated, patients are also bridged to heart transplantations from VA ECMO.

**Routine care immediately after ECPR:**

- Distal perfusion cannula should be inserted as soon as time allows and maximum before 4 hrs post cannulation (e.g. post coronary angiography / PCI)
- Mechanical ventilation with PEEP ≥ 10 cm H_2_O to prevent pulmonary oedema, reduce minute ventilation as the patients have reduced pulmonary blood flow while on VA ECMO.
- Establish right-sided arterial line (radial or brachial) and titrate vasopressors to MAP target
- 12 Lead ECG
- Monitor for early access insufficiency (ECMO circuit line chatter)
- Monitor for loss of pulsatility, access insufficiency (ECMO circuit line chatter)
- If access insufficiency occurs- bedside check for aortic insufficiency with point of care cardiac ultrasound. Evaluate for LV distension- distended, non-ejecting LV with “empty RV” (Aortic regurgitation may not appear severe due to rapid equalization of pressures)
- Perform abdominal FAST scan for obvious source of abdominal bleeding
- Fluid bolus to account for dilution post initiation of extracorporeal support
- Patients in refractory arrhythmias may have another attempt at electrical cardioversion after several minutes of extracorporeal support. Restoring coronary perfusion pressure and improving perfusion and acid-base may favour successful cardioversion at this point. However, coronary ischaemia (if present) needs to be addressed as well as other reversible factors such as hyperkalaemia and/or if relevant hypothermia. Multiple attempts should therefore be avoided unless those factors are resolved.
- Restoration of a potentially perfusing rhythm increases the likelihood of LV ejection and thus reduces risks of LV distension and pulmonary oedema.
- Emergency coronary angiography should be strongly considered for all ECPR patients independent of age and presenting rhythm in the absence of an obvious alternate non-cardiac cause.

**LV distension in cardiac cath lab**

ECPR patients are at high risk of developing LV distension syndrome.

Patients to be considered for LV venting are:

- Patients that remain with a pulse pressure less than 10mmg at the end of the procedure

OR

- patients that fit the clinical syndrome with high LVEDP, low ejection fraction and early pulmonary oedema

LV venting options include

- Surgical trans-apical LV vent
- Impella CP
- Transaortic Pig-tail venting

Alternate causes of loss of pulsatility need to be considered in parallel such as hypovolaemia and bleeding.

Optimise medical management for LV distention

- Medical management is appropriate for patients at risk or with mild forms of LV distension where recovery of LV function is anticipated. It is not a long-term strategy and prevents extubation.
- Lower MAP to 60-70 mmHg
- High PEEP (15 to 25 cm H_2_0)
- Inotropic support for maintenance of pulsatility. Note: inotropes may exacerbate LV distension if LV is non-responsive to inotropes by primarily increasing RV output
- Volume state reduction (fluid removal) if blood pressure and venous pressures are adequate
- Altering VA ECMO flow. Note: In particular, where RV systolic function is better than LV systolic function reducing pulmonary blood flow by increased ECMO blood flow can be effective. The afterload, however, needs to be controlled to MAP target with systemic vasodilators as required.
- Intra-aortic balloon pumping (IABP) may provide additional afterload reduction in patients at risk of LV distension. Previously inserted IABP support should be maintained following VA ECMO initiation in patients at risk for LV distension in the absence of IABP complications like leg ischaemia or bleeding.

**CT Imaging post ECPR**

Routine CT imaging is performed in all ECPR cases as soon as practical. If the cause of the cardiac arrest is unclear, the scan will occur after the cannulation in the Emergency & Trauma Centre – otherwise after cardiac cath lab on the way to ICU.

**Targeted temperature management**

Targeted temperature management of 36.0°C can generally be achieved by use of the oxygenator heat exchanger. If the patient presents already below this temperature then rewarm at 0.25°C per hour until 36.0°C is achieved. It is important to note that the ongoing temperature may need to be set below or above 36°C to achieve a body core temperature of 36°C. The body core temperature is easily taken with the use of bladder temperature probe catheters (on the Cardiohelp console the circuit temperature may serve as a surrogate). The targeted temperature should be maintained strictly for a period of 24 hours post cardiac arrest.

**Initial ICU Management**

Respiratory

Reduce minute ventilation (e.g. PCV with PC of 10 cm H_2_O, rate of 10/minute). Titrate PEEP as required. High PEEP may assist in attenuating LV distension in left ventricular dysfunction and reduce the risk of pulmonary oedema. It may also improve LV afterload. Minimum FiO_2_ on the ventilator should be 0.5.

Cardiovascular

Noradrenaline should be titrated for a MAP of 65 mmHg. If an inotrope is required to achieve pulsatility, adrenaline infusion allows for immediate support. TTE should be performed to assess cardiac function and check for regurgitant lesions. LV distension is a common problem in this patient group, generally develops early and should be addressed promptly with medical management and LV venting options as indicated.

Neurological

Sedation may be required if there are signs of awakening. Morphine and midazolam infusions are commonly used, propofol may be added. The former is preferred if blood pressure is unstable and vasopressors are required.

Renal / Electrolytes

Frequent monitoring and appropriate correction of electrolytes (K+, Mg2+, PO_4_^3-^) is required. Post cardiac arrest transient AKI is common. Renal replacement, if required, should be provided with continuous clearance via the ECMO circuit.

Endocrine

Hyperglycaemia is common and should be treated according to the usual ICU Insulin infusion protocol. Avoid calcium infusions to correct asymptomatic hypocalcaemia in patients with suspected hypoxic ischaemic encephalopathy.

Haematology

In post cardiac arrest patients on ECMO it is not uncommon for the patient to be coagulopathic with an increased bleeding risk (especially if the patient undergoes PCI covered with dual antiplatelet agents). Most will have received heparin as a bolus during cannulation with the cannula flushes and in the cath lab. GP IIb/IIIa antagonists in addition should be used with caution. Once it is clear that the patient is not actively bleeding, heparin infusion may commence, with the APTT targeted as per the usual anticoagulation guideline.

**Prognostication after cardiac arrest**

The assessment of neurological recovery after ECPR does not principally differ from other cardiac arrest patients and is generally undertaken at 96 hours (>48 hours after cessation of sedation) and is based largely on the clinical neurological examination.

However, it is important to note that some patients will have progressive multi-organ failure in the first 24-48 hrs and may not be supportable with ECMO. Further, patients that do require sedation with recurrent access insufficiency on waking can be challenging to manage. Early team discussion including potential reconfiguration may be considered to avoid ongoing sedation.

In ECPR patients, the assessment will follow the standard Alfred ICU pathways. Organ donation may be considered in patients who meet the criteria for brain death or, if severe neurological injury is diagnosed, then donation after circulatory death (DCD) may be considered, as this is likely to occur shortly after the withdrawal of VA ECMO support.

**Loss of Pulsatility**

Causes of loss of pulsatility on the arterial line trace include

- Artifact
- Inadequate preload (e.g. excessive VA ECMO flow, haemorrhage)
- Mechanical obstruction (e.g. tamponade, tension pneumothorax, RVOT, LVOT obstruction, pulmonary embolism). Intrathoracic bleeding causing cardiac compression is a common cause immediately following intrathoracic surgery.
- Arrhythmias
- LV failure, poor LV contractility e.g. ischaemia / infarction
- Excessive afterload: typical MAP target 65-70 mmHg
- Valvular regurgitation: Aortic or Mitral regurgitation

Consequences of loss of pulsatility

- Thrombosis
- Stagnating or low flow across the left ventricle and aortic root
- On echocardiography this may manifest as spontaneous echo contrast (SEC) which may progress to manifest thrombus
- Massive intracardiac thrombosis: generally lethal
- Thromboembolism with potential cerebral, limb, kidney or gut ischaemia / infarction.
- Increased left ventricular filling pressure/High LV load
- Loss of left ventricular ejection (forward flow) can increase the risk of pump driven LV distention (Left Ventricular Failure). Avoiding high LV afterload is a primary concern during VA ECMO.
- Impaired coronary perfusion and further ischaemic damage to the myocardium
- Mitral regurgitation with resultant left atrial hypertension, pulmonary oedema, pulmonary capillary rupture and haemorrhage.

Assessment

Clinical assessment should consider following causes:

- Artifact: check for damping of waveform and palpable peripheral pulses. Replace arterial line if required
- Arrhythmia: exclude non-perfusing rhythms
- Console: check flow and pump speed setting. Assess for signs of Access Insufficiency and perform dynamic assessment
- Hb: exclude clinically apparent bleeding (retroperitoneal, pleural, GIT), downward trend on ABG or FBE
- Tension pneumothorax: palpate, Auscultate and consider CXR
- Tamponade: bedside Echo, measure CVP and assess for Access Insufficiency
- Pulmonary oedema: ETT (frothy secretions +- haemorrhage) or on CXR.
- Echocardiography looking for features of: Tamponade, Right heart failure, Left heart failure, valvular regurgitation,
- Note: In ECPR and more severe forms of acute cardiac failure, it is common to lose pulsatility immediately after commencing VA ECMO. As VA ECMO is always combined with cardiac ultrasound assessment, in this setting, it is not necessary to repeat cardiac ultrasound examinations.
- Additional eFAST assessment may be performed concurrently where appropriate (e.g. assessing for haemothorax / substantial intraperitoneal fluid).

Management

Management depends on the underlying cause found on assessment, and may include:

- Inadequate preload: consider volume loading (including blood / blood products). NOTE: LV failure with LV distention syndrome is usually associated with a “sucked down” RA and IVC. Volume loading will not correct this problem. The “need” for volume loading in VA ECMO should always prompt a search for LV failure and bleeding.
- Mechanical obstruction: usually surgical management with adjunctive medical management (e.g. volume loading as temporising measure; correcting coagulopathy preoperatively in tamponade)
- Poor contractility: treat underlying cause (e.g. angiography for ischaemia) and optimise temperature, acidosis, pO2 and pCO2.
- Arrhythmia: aim for K^+^ > 4.5 and Mg^++^ > 1 (or higher) in ventricular arrhythmias and consider amiodarone / direct current cardioversion (DCR). β blockade may be instituted after senior consultation. Consider cardiology referral.
- RV / LV failure: consider inodilators or constrictors (e.g. milrinone, adrenaline) as dictated by MAP, with pulmonary vasodilators (e.g. iNO) if required.
- Excessive afterload: review MAP targets and consider inodilators / vasodilators. PEEP application. Aggressive afterload control cannot be underestimated in these situations.
- In the absence of pulmonary oedema, with adequate systemic perfusion and MAP, managing inadequate preload and / or excessive afterload may resolve the loss of pulsatility.

Measures to combat the consequences of loss of pulsatility

- Increased anticoagulation, particularly in the setting of SEC or thrombus, should be considered in the context of other clinical priorities (e.g. haemostasis).
- LV decompression

**Figure A1. Distribution of time-weighted mean pulse pressure in the first 24 hours between loss of pulsatility and pulsatile groups**

Shown are the filled density curves of time-weighted mean pulse pressure between loss of pulsatility and pulsatile group.

**Table A1. Factors associated with mortality using number of hours of loss of pulsatility as an independent variable**

| **Characteristic** | **Univariable analysis** | | **Multivariable analysis** | |
| --- | --- | --- | --- | --- |
|  | **OR (95% CI)** | **p-value** | **Adj OR (95% CI)** | **p-value** |
| Duration of loss of pulsatility (hrs) | 1.21 (1.01-1.34) | <0.001 | 1.17 (1.05-1.30) | 0.004 |
| Age (yrs) | 0.99 (0.97-1.01) | 0.50 | 1.00 (0.98-1.03) | 0.87 |
| Sex | | | | |
| Female | Ref |  | Ref |  |
| Male | 1.00 (0.54-1.85) | 0.99 | 0.77 (0.35-1.68) | 0.51 |
| APACHE-III-J | 1.00(0.996-1.01) | 0.44 | - |  |
| Location of cardiac arrest | | | | |
| OHCA | Ref |  | Ref |  |
| IHCA | 0.42 (0.24 -0.73) | 0.002 | 0.72 (0.34-1.53) | 0.39 |
| First monitored rhythm | | | | |
| PEA/Asystole | Ref |  | Ref |  |
| VT/VF | 0.81 (0.47-1.40) | 0.33 | 0.37 (0.17 - 0.77) | 0.01 |
| Diagnostic group | | | | |
| Non-AMI | Ref |  | Ref |  |
| AMI | 1.77 (1.03-3.05) | 0.04 | 2.19 (1.05 – 4.56) | 0.04 |
| Arrest to ECMO time (min) | 1.02 (1.01-1.03) | <0.001 | 1.01 (1.00-1.02) | 0.04 |
| VIS* | 1.005 (1.002-1.007) | <0.001 | 1.004 (1.002-1.007) | <0.001 |
| pH^ⴕ^ | 0.06 (0.02-0.22) | <0.001 | - |  |
| PaO_2_ (mmHg) | 1.00(1.00-1.002) | 0.36 | - |  |
| PaCO_2_ (mmHg) | 1.02 (1.01-1.03) | 0.004 | - |  |
| Lactate (mmol/L)^ⴕ^ | 1.17 (1.09-1.25) | <0.001 | - |  |
| Base Excess^ⴕ^ | 0.94 (0.90-0.97) | 0.001 | - |  |
| LV Vent | | | | |
| No | Ref |  |  |  |
| Yes | 0.86 (0.29-2.56) | 0.78 | - |  |
| VAD or Heart Transplantation | | | | |
| No | Ref |  |  |  |
| Yes | 0.06 (0.01-0.46) | 0.007 | - |  |
| Continuous Renal Replacement Therapy | | | | |
| No | Ref |  |  |  |
| Yes | 1.62 (0.91-2.87) | 0.10 | - |  |

*Maximum VIS in the first 24 hours is included in the model.

^ⴕ^The blood gas parameters were not included in the final model due to significant correlation with the arrest-to-ECMO-time.

OR- odds ratio, CI- confidence interval, APACHE- Acute Physiology and Chronic Health Evaluation, OHCA- Out of hospital cardiac arrest, IHCA- In-hospital cardiac arrest, PEA- Pulseless electrical activity, VT/VF- Ventricular tachycardia/ ventricular fibrillation, AMI- Acute myocardial infarction, ECMO- Extracorporeal membrane oxygenation, VIS- Vasoactive inotropic score, LV- Left ventricle, VAD- Ventricular assist device.

The multivariable logistic regression model showed adequate discrimination and calibration: area under receiver operating characteristic curve=0.78, Hosmer-Lemeshow Goodness of Fit χ2(df 8)= 7.55, p=0.48.

**Supplementary Table A2. Factors associated with in-hospital mortality using time-weighted mean pulse pressure as independent variable**

| **Characteristic** | **Univariable analysis** | | **Multivariable analysis** | |
| --- | --- | --- | --- | --- |
|  | **OR (95% CI)** | **p-value** | **Adj OR (95% CI)** | **p-value** |
| Pulse pressure (mmHg) | 0.94 (0.91-0.96) | <0.001 | 0.95 (0.93-0.98) | <0.001 |
| Age (Yrs) | 0.99 (0.97-1.01) | 0.50 | 1.00 (0.98-1.03) | 0.81 |
| Sex | | | | |
| Female | Ref |  | Ref |  |
| Male | 1.00 (0.54-1.85) | 0.99 | 0.75 (0.34-1.65) | 0.48 |
| APACHE-III-J score | 1.00(0.996-1.01) | 0.44 | - |  |
| Location of cardiac arrest | | | | |
| OHCA | Ref |  | Ref |  |
| IHCA | 0.42 (0.24 -0.73) | 0.002 | 0.74(0.34-1.60) | 0.44 |
| First monitored rhythm | | | | |
| PEA/Asystole | Ref |  | Ref |  |
| VT/VF | 0.81 (0.47-1.40) | 0.33 | 0.37 (0.17-0.81) | 0.01 |
| Diagnostic Group | | | | |
| Non- AMI | Ref |  | Ref |  |
| AMI | 1.77 (1.03-3.05) | 0.04 | 1.87 (0.87-4.03) | 0.11 |
| Arrest to ECMO time (min) | 1.02 (1.01-1.03) | <0.001 | 1.01 (1.00-1.02) | 0.02 |
| VIS* | 1.005 (1.002-1.007) | <0.001 | 1.003 (1.001-1.006) | 0.01 |
| pH^ⴕ^ | 0.06 (0.02-0.22) | <0.001 |  |  |
| PaO_2_ (mmHg) | 1.00(1.00-1.002) | 0.36 | - |  |
| PaCO_2_ (mmHg) | 1.02 (1.01-1.03) | 0.004 | - |  |
| Lactate (mmol/L)^ⴕ^ | 1.17 (1.09-1.25) | <0.001 | - |  |
| Base Excess^ⴕ^ | 0.94 (0.90-0.97) | 0.001 | - |  |
| LV Vent | | | | |
| No | Ref |  |  |  |
| Yes | 0.86 (0.29-2.56) | 0.78 | - |  |
| VAD/ Heart Transplantation | | | | |
| No | Ref |  |  |  |
| Yes | 0.06 (0.01-0.46) | 0.007 | - |  |
| Continuous Renal Replacement Therapy | | | | |
| No | Ref |  |  |  |
| Yes | 1.62 (0.91-2.87) | 0.098 | - |  |

*Maximum VIS in the first 24 hours is included in the model.

^ⴕ^The blood gas parameters were not included in the final model due to significant correlation with the arrest-to-ECMO-time.

OR- odds ratio, CI- confidence interval, APACHE- Acute Physiology and Chronic Health Evaluation, OHCA- Out of hospital cardiac arrest, IHCA- In-hospital cardiac arrest, PEA- Pulseless electrical activity, VT/VF- Ventricular tachycardia/ ventricular fibrillation, AMI- Acute myocardial infarction, ECMO- Extracorporeal membrane oxygenation, VIS- Vasoactive inotropic score, LV- Left ventricle, VAD- Ventricular assist device.

The multivariable logistic regression model showed adequate discrimination and calibration (area under receiver operating characteristic curve=0.80, Hosmer-Lemeshow Goodness of Fit χ2(df=8)= 2.84, p=0.94.

**Figure A2. Predicted probability of in-hospital mortality across time-weighted mean pulse pressure, adjusted for covariates**

Ribbon plot of covariate-adjusted predicted probability of in-hospital mortality across time-weighted mean pulse pressure (mmHg) derived from the multivariable model using margins command shown in Table A1. The upper and lower borders of the ribbon plot show the 95% confidence interval.
